# *Uncaria nervosa Elme*r, a new herbal source for betulinic acid and ursolic acid: Metabolites profiling, isolation, and in vitro cytotoxicity studies against T47D breast cancer

**DOI:** 10.12688/f1000research.152293.1

**Published:** 2024-08-15

**Authors:** Noveri Rahmawati, Nor Hadiani Ismail, Fatma Sri Wahyuni, Dachriyanus Hamidi

**Affiliations:** 1 Doctoral Program, Faculty of Pharmacy, Universitas Andalas, Padang, West Sumatra, Indonesia; 2 Faculty of Applied sciences, Universiti Teknologi MARA, Shah Alam, Selangor, 68100, Malaysia; 3 Faculty of Pharmacy, Universitas Andalas, Padang, West Sumatra, 25166, Indonesia

**Keywords:** Isolation; cytotoxicity; LCMS/MS; T47D; Uncaria nervosa Elmer, MZMine, SIRIUS

## Abstract

**Background:**

*Uncaria nervosa* Elmer is an Indonesian herbal plant that is traditionally used for breast cancer. The results of phytochemical screening contained alkaloids, flavonoids, and terpenoids in the ethanol extract of this plant. Based on literature searches, reports regarding the bioactive compounds responsible for breast cancer have not been found. Further research is needed to understand the potential of *Uncaria nervosa* Elmer as a breast cancer treatment and to identify the specific compounds responsible for its effects

**Methods:**

This study aims to determine the metabolite profiling of ethanol extract, the isolation, characterization of bioactive compounds, and their bioactivity in T47D breast cancer cells. The research began by extracting the leaves by maceration using 70% ethanol, and then solid phase extraction was carried out using the solid phase extraction (SPE) method. In this study, the sorbent used was polyamide. The extract was analyzed using a tandem analysis technique based on LCMS using the MZmine and SIRIUS platforms. Isolation was carried out using column chromatography, and preparative recycling HPLC. Bioactive compounds were characterized using UV, HPLC, NMR, and 2D NMR, as well as bioactivity tests using the MTT method.

**Results:**

The results show that the extract contained N-[(1,3-dimethyl-2,6-dioxo-7-prop-2-ynylpurin-8-yl) amino] formamide, N-(3-phenylbutyl)hexan-2-amine, 1,1-Dichloro-1-nitrosopropane, ceratodictyol, betulinic acid, ursolic acid, 7-methyl-N-[6-[(7-methyl-6-oxooctanoyl) amino] hexyl]-6-oxononanamide, Nervisterol and 3,5,10-tris (acetyloxy)-2-hydroxy-4,14,16,16-tetramethyl-8-methylidene-13-oxo-15oxatetracyclo [9.4.1.0 ^1^, ^14^.0 ^4^, ^9^] hexadecan-7-yl 3-phenylprop-2-enoate. The ethanol extract of *Uncaria nervosa* Elmer leaves contains nine compounds consisting of alkaloids, terpenoids, and fatty acid. The bioactive compounds that were successfully isolated were betulinic acid, and ursolic acid, with IC _50_ values of ˃100 and 14,70±4,50 μg/ml, respectively. These compounds were reported in this plant for the first time.

**Conclusion:**

Betulinic acid, and ursolic acid have been successfully isolated from leaves *Uncaria nervosa* Elmer, and ursolic acid have moderate cytotoxic activity on T47D breast cancer cells.

## Introduction

Breast cancer is cancer that forms in breast cells. This disease occurs in men and women, but women are more often affected by this disease. Breast cancer usually starts in the ductal carcinoma or lobular carcinoma of the breast. This disease spreads through the lymphatic system or bloodstream. Breast cancer is the most common cancer in women throughout the world, with incidence rates varying in each country. According to the World Health Organization (WHO), there were an estimated 2.3 million new cases of breast cancer diagnosed worldwide in 2020 ( Sung et al., 2021). One of the current treatments for breast cancer is chemotherapy. These side effects will be different for each person. The type of drug, dosage, length of time using the drug and the medical history of each individual are factors that greatly influence the side effects that will occur. The cancer treatment continues to develop rapidly and drug development continues, but this disease is still an evolutionary process. Cancer cells can adapt to the treatment given. Many studies have shown that natural products can kill cancer cells and also limit their proliferation by targeting several target molecules and genes in cancer cells ( Newman and Cragg, 2020; Zhu et al., 2022).

Plants are a source of medicinal compounds, due to their ability to synthesize bioactive compounds, especially secondary metabolites ( Joshua et al., 2020). *Uncaria nervosa* Elmer, commonly known as “Akar Kaik Kaik” is a plant species belonging to the genus *Uncaria* within the family Rubiaceae. This botanical species is indigenous to Southeast Asia, particularly found in regions such as Indonesia, Malaysia, and the Philippines. *U. nervosa* is renowned for its medicinal properties and has been an integral part of traditional herbal medicine practices in the region for centuries. *U. nervosa* is a woody vine characterized by its distinctive climbing habit, with slender stems that can reach significant lengths as they twine around trees and other support structures in their natural habitat. The leaves are opposite, elliptical to lanceolate in shape, and possess prominent veins. The plant produces small, tubular flowers with whitish or yellowish hues, which eventually give rise to small fruits containing seeds ( Anonim, n.d.; Maulina et al., 2019).

*U. nervosa* has been revered for its diverse medicinal properties and has been used by indigenous communities for various health purposes. In traditional herbal medicine, different parts of the plant, including the leaves, stems, and roots, are utilized to prepare decoctions, infusions, or poultices to address ailments such as fever, inflammation, digestive disorders, and respiratory conditions. Additionally, *U. nervosa* has been valued for its purported benefits in promoting overall wellness and vitality. The pharmacological activities of *U. nervosa* are attributed to its rich phytochemical composition, which includes alkaloids, flavonoids, phenolic compounds, tannins, and triterpenoids. Triterpenoid is a terpenoid, often having a pentacyclic or tetracyclic structure ( Hill and Connolly, 2020; Ling et al., 2022; Noushahi et al., 2022). Pentacyclic triterpenes consist of five isoprene units which are classified into lupane, oleanane, and ursane types ( El-Dawy et al., 2022; Kaps et al., 2021; Yanlin Li, Jing Wang, Linyong Li, Wenhui Song, Min Li, Xin Hua, Yu Wang, Jifeng Yuan, 2023). These compounds have a wide spectrum of pharmacological properties including, cardiovascular activities, antioxidant, antimicrobial, antiviral, antidiabetic, anti-inflammatory, anti-ulcerogenic, anti-obesity, anti-aging, analgesic, immunomodulatory, hypolipidemic, neuroprotective, hepatoprotective, and anticancer ( Adham et al., 2020; Chooluck et al., 2021; Wang et al., 2020). Research on the isolation and cytotoxic activity of *U. nervosa* has not been widely explored. The cytotoxic activity of ethanol extract of *U. nervosa* leaves on T47D breast cancer cells has been reported by previous researchers where the IC _50_ value was obtained at 64.42 μg/ml ( Rahmawati et al., 2023). In this study, the metabolites profiling of ethanol extract was determined using LCMS/MS, isolation using column chromatography and preparative recycling HPLC and cytotoxic testing of T47D breast cancer cells using the MTT method.

## Methods

*U. nervosa* Elmer plant obtained from the Kampar Forest, Riau, Indonesia. The plant was identified in the ANDA Herbarium, Andalas University, Padang with voucher number NR-04. The chemicals used are ethanol (Merck 1.00983.2500), methanol (Merck 1.06007.4000), acetonitrile (Merck, 1000304000), n-hexane (Merck 1.04367.2500), ethyl acetate (Merck 1.09623.1000), butanol (Merck 1.01990.1000), DMSO, cells T47D, phosphate buffered saline (PBS) (Sigma, P5119), culture media, 20% MTT solution 3-(4,5-dimethylhiazolyl-2,5-diphenyltetrazolium bromide (Merck, M2003).

The equipment used were LCMS/MS (Thermo Scientific ^TM^ Orbitrap Fushion ^TM^ mass spectrometer), recycling preparative HPLC (Japan analytical industry ^TM^), column Jaigel-ODS-AP.SP-120-15 20x250 mm, liquid nitrogen tank, 96 hole microplate, hemocytometer, biosafety cabinet (Faster), micropipettes (10, 20, 200, 1000 μl), culture flask, 1.5 ml centrifuge tube, inverted/phase contrast microscope (Olympus), centrifugator, CO2 incubator, spectrophotometric microplate reader (Thermo), ELISA reader, Laminar Air Flow and FACS-Calibur.

### Crude extraction and liquid-liquid fractionation

*U. nervosa* (1.932 g) leaves were macerated using the 2000 ml ethanol (Merck 1.00983.2500). The ethanol extract was fractionated using a separating funnel starting with 500 ml n-hexane (Merck 1.04367.2500), 500 ml ethyl acetate (Merck 1.09623.1000), and 500 ml butanol (Merck 1.01990.1000) as solvents. The three fractions were thickened using a rotary evaporator.

### Metabolites profiling of ethanol extract

**Ultra-High-Performance Liquid Chromatography (UHPLC)**

The ethanol extract (20 mg) was cleaned up using SPE-polyamide and obtained 5.7 mg (28.5% recovery). SPE is carried out by rinsing using 3 ml methanol (Merck 1.06007.4000) and a conditioning process with water which aims to eliminate interference in the sample (2:98, v/v). The sample was filtered with a 0.45 μm UHPLC filter, then placed in an HPLC vial. The crude was transferred to the Eppendorf tube and dissolved in 1 mL of solvent mixture (80% methanol and 20% water). The tube was sonicated and the solution was filtered (0.22 μm) then put into an HPLC bottle. The stationary phase used was a Luna Omega C18 column (100 x 2.1 mm, 1.6 μm) with a diode array detector (DAD). The sample injected was 1 μL, the column temperature was 30°C, and the system flow rate was 0.28 mL/minute. Elution was carried out using deionized water (% A) and the organic solvent, acetonitrile (Merck, 1000304000), (% B) and solvent system: 0-30 minutes (5-100% B) and 30-40 minutes (100% B) ( Ammar et al., 2024).

**Liquid Chromatography Mass Spectrometry (LCMS)**

LCMS analysis at a temperature of 30 °C. The sample (1000 ppm), was dissolved in a mixture of methanol and water, then injected 1 μL and the flow rate was set to 0.2 mL/min. LCMS grade water (% A) and methanol (% B) were used in the analysis, with elution systems of 0-30 minutes (10-100% B) and 30-35 minutes (100% B). The samples were filtered using positive ionization mode and analyzed using multistage mass spectrometry (MS/MS) data analysis.

### Isolation and elucidation of bioactive compound

The ethyl acetate fraction was weighed as much as 20 g, preabsorbed using silica gel and then put into a chromatography column. The fraction was eluted using a step gradient polarity system solvent, using n-hexane 250 ml (Merck 1.04367.2500), ethyl acetate 250 ml (Merck 1.09623.1000), and methanol 250 ml (Merck 1.06007.4000). There were 11 sub-fractions obtained and the one that was continued for purification was sub-fraction E (HE 5:5). Purification was carried out using recycling preparative HPLC with 1000 ml acetonitrile (Merck, 1000304000) and distilled water as eluents (98:2) ( Ammar et al., 2024). Elucidation of the structure of the isolated compound was carried out using a mass spectrometer, 1H- and 1D and 2D-NMR.

### Cytotoxic assay against T47D cells

**Cell lines and culture condition**

T47D breast cancer cells are a collection of the Cancer Chemoprevention Research Center (CCRC) Gajah Mada University (UGM) Indonesia. T47D cancer cells were taken from the liquid nitrogen tank in a sterile manner, and then the cancer cells in a cyro tube were thawed over a wet bath at a temperature of 37°C for 2-3 minutes. Cancer cells were transferred into a falcon tube containing 9 mL of DMEM and centrifuged at 2000 rpm (10 minutes). The supernatant was removed by pipetting it, leaving a layer of pellets. Next, 2 mL of medium was added to the pellet layer and transferred into a flask, then incubated in a 5% CO _2_ incubator at 37°C for 3-4 hours. The flask was observed under an inverted microscope to see whether the cells adhered to the bottom of the flask and formed a monolayer. The growth medium was changed once every two days, and when the number of cells in the flask reached 70-85% confluent, cell sub-culture was carried out.

**Cell seeding**

Cell of suspension (180 μL) was made in cell suspension medium, and put into each well except the wells in the first and last columns and the first and last row. The first and last columns, as well as the first and last rows, are blanks, which only contain 200 μL of medium, while the second and seventh columns are controls, which contain 200 μL of cell suspension. Incubate in a 5% CO _2_ incubator at 37°C for 24 hours.

### Cytotoxicity assay

Determination of cytotoxic activity was carried out on betulinic acid, ursolic acid and doxorubicin. The 96-well plate, which contains 180 μL of cell suspension and has been incubated for 24 hours, is removed from the incubator. The test solution (20 μL) for each concentration of 0.1-67 μg/mL was transferred into each well except the control well and blank well to obtain a test solution with a concentration of 100, 10, 1, and 0.1 μg/mL. The 96-well plate was again incubated for 48 hours in a 5% CO _2_ incubator at 37°C. After that, the medium was discarded, then 100 μL of PBS (Sigma, P5119) was added to each well, and the PBS was removed by pipetting. 100 μL of 0.5 mg/mL MTT (Merck, M2003) solution was pipetted into each well and incubated for 4 hours in a 5% CO _2_ incubator at 37°C. After 4 hours, a purple precipitate of formazan crystals will form. Then the cell condition is observed using an inverted microscope. The MTT reagent is discarded, so that what remains is only a purple precipitate of formazan crystals. The precipitate in each well was dissolved in 100 μL of DMSO, and its absorbance was measured using an ELISA reader spectrophotometer at λ 550 nm. Based on the absorbance value between the sample and control cells, the % viability value can be determined. The data is processed using graphPad prism software to obtain the IC _50_ value. The cytotoxicity results for the chemicals strong cytotoxicity: IC _50_<4 μg/mL (or IC _50_<10 μM); moderate cytotoxicity: 4 μg/mL< IC _50_<20 μg/mL (or 10 μM< IC _50_<50 μM); low cytotoxicity: 20 μg/mL< IC _50_<100 μg/mL (or 50 μM< IC _50_<250 μM); no cytotoxicity: IC _50_>100 μg/mL (or IC _50_>250 μM) ( Bonsou et al., 2022).

### Statistical analysis

Data analysis of extract metabolites profiles and structure elucidation in the form of tables, graphs and images. IC _50_ values of samples and controls were determined using statistical analysis GraphPad Prism Software V 9.0.0 (GraphPad Software LLC).

## Results

### Metabolites profiling of ethanol extract

**Figure 1.**
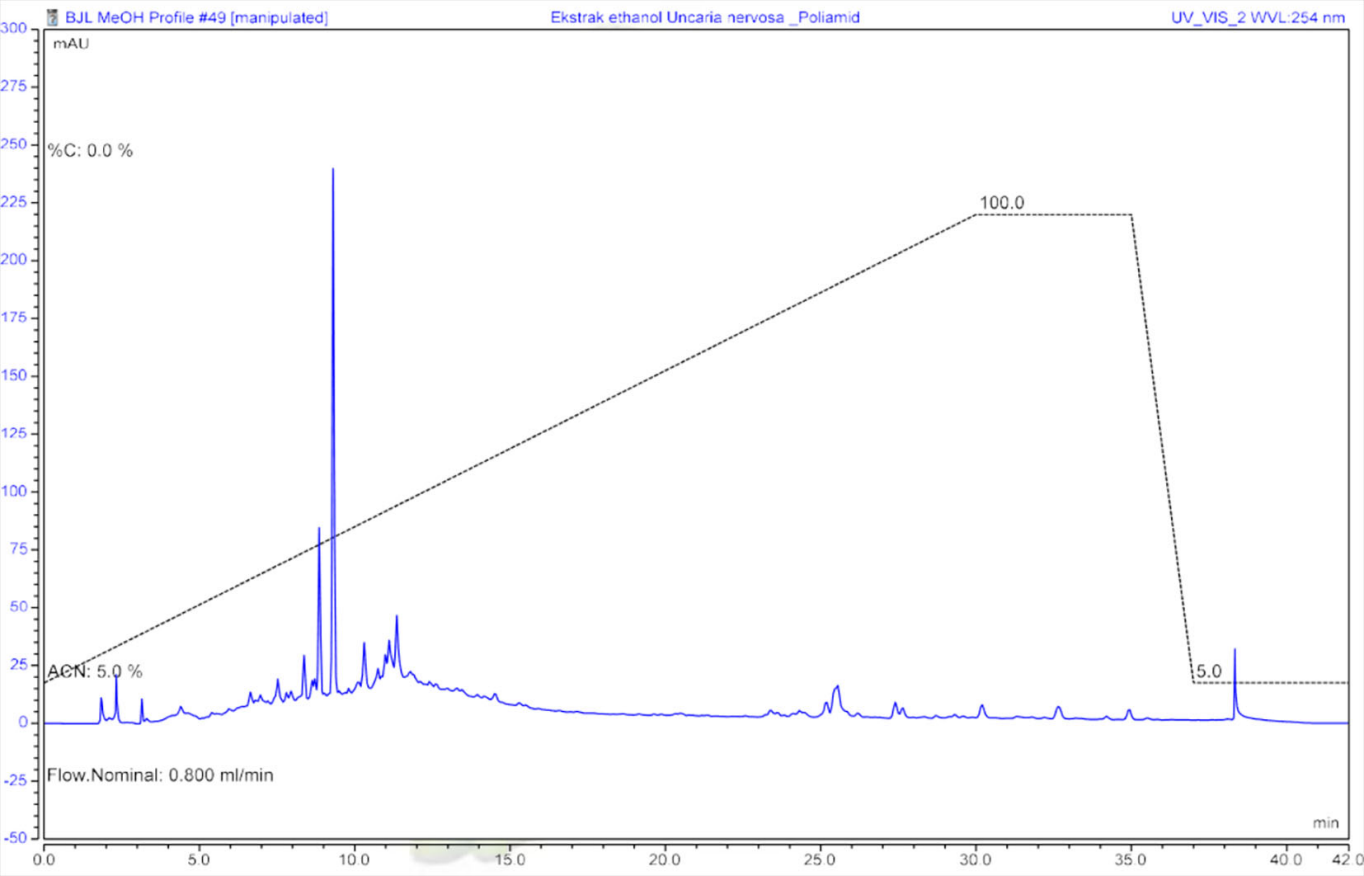
Chromatogram of ethanol extract of *U. nervosa* leaves at UV 254 nm.

**Figure 2.**
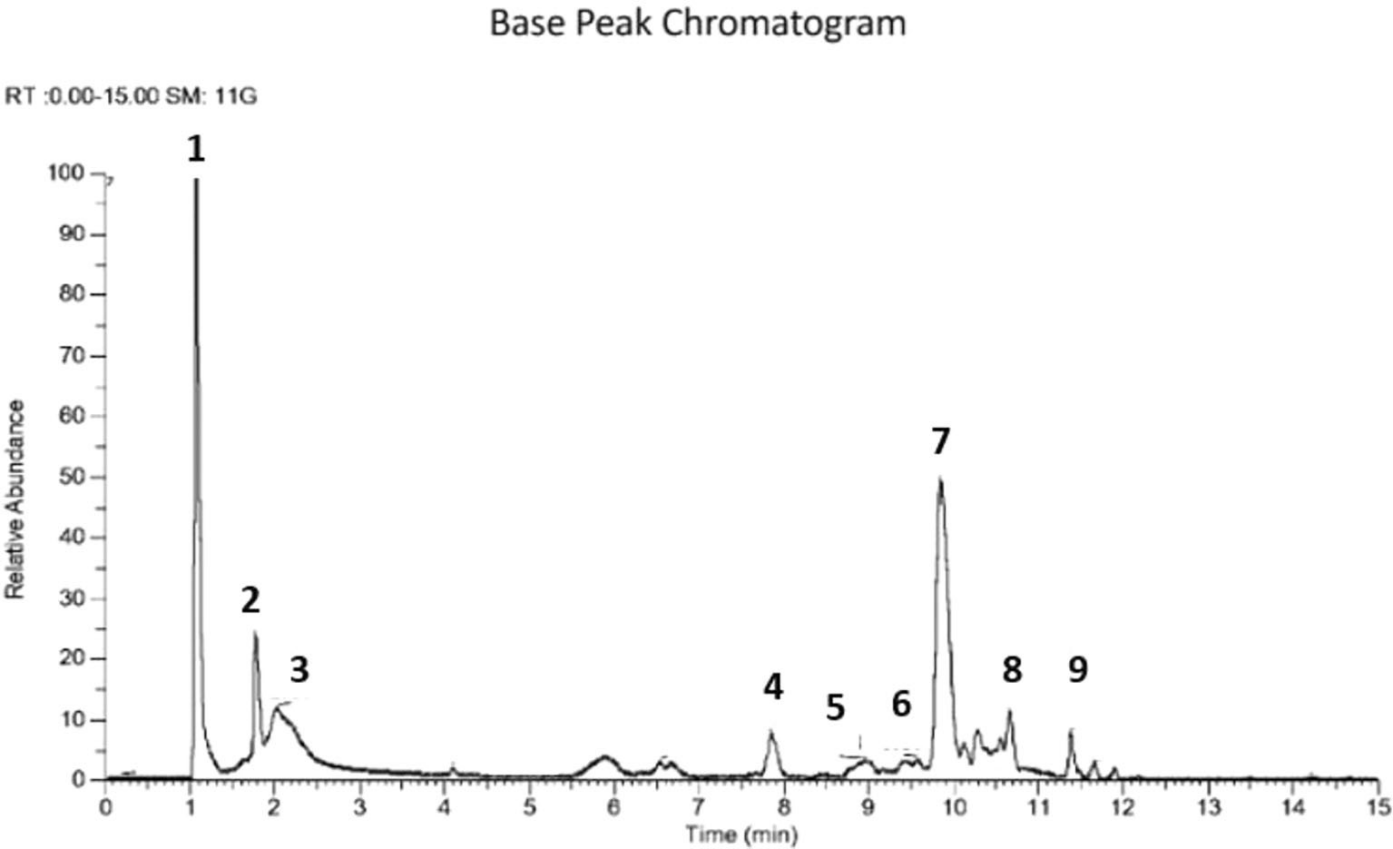
Base peak chromatogram of ethanol extract of the leaves of *U. nervosa.*

Determination of the metabolite profile of ethanol extract begins with the clean-up process of ethanol extract using the solid phase extraction (SPE) method. After the clean-up is complete, the extract is prepared for chromatogram determination using UHPLC. The chromatogram profile was monitored under a UV 254 lamp ( Figure 1). The extract was prepared to determine the metabolite profile using LCMS. Raw data obtained from the instrument is processed by carrying out data conversion, data processing and metabolite annotation using the Sirius platform. In the ethanol extract, it is predicted that there are 9 compounds ( Figure 2, Table 1), contained N-[(1,3-dimethyl-2,6-dioxo-7-prop-2-ynylpurin-8-yl) amino] formamide, 4-decylaniline, robinin, ceratodictyol, betulinic acid, ursolic acid, 7-methyl-N-[6-[(7-methyl-6-oxooctanoyl) amino] hexyl]-6-oxononanamide, hexadecanamide and 3,5,10-tris (acetyloxy)-2-hydroxy-4,14,16,16-tetramethyl-8-methylidene-13-oxo-15oxatetracyclo [9.4.1.0 ^1^, ^14^.0 ^4^, ^9^] hexadecan-7-yl 3-phenylprop-2-enoate.

**Table 1.** Predicted compounds from the ethanol extract of the leaves of *U. nervosa.*

Peak	RT (min)	Compound identification	Molecular formula	Precursor ion (m/z)	MS/MS fragmentation	Group	Sirius similarity (%)
1	1.09	N-[(1,3-dimethyl-2,6-dioxo-7-prop-2-ynylpurin-8-yl) amino] formamide	C _11_H _12_N _6_O _3_	277.10	139.01, 121.05	Alkaloid	61
2	1.76	N-(3-phenylbutyl) hexan-2-amine	C _16_H _27_N	234.22	205.18, 204.17, 190.15, 177.15, 163.13, 176.14, 162.12, 149.11, 148.11	Alkaloid	99
3	2.09	1,1-Dichloro-1-nitrosopropane	C _3_H _5_Cl _2_NO	141.98	113.96, 100.95, 97.96	Fatty acid	99
4	7.85	Ceratodictyol	C _19_H _38_O _4_	331,28	257.24, 239.23, 221.22	Fatty acid	100
5	9.00	Betulinic acid	C _30_H _48_O _3_	455.33	453.77, 353.67, 99.46	Triterpenoid	70
6	9.57	Ursolic acid	C _30_H _48_O _3_	455.65	450.54, 407.67, 345.45, 316.83, 187.46	Triterpenoid	100
7	9.86	7-methyl-N-[6-[(7-methyl-6-oxooctanoyl) amino] hexyl]-6-oxononanamide	C _25_H _46_N _2_O _4_	439.35	394.35, 270.22, 269.22, 244.21, 242.19, 243.20, 241.19	Diterpenoid	100
8	10.66	Nervisterol	C _30_H _48_O	425.37	407.36, 131.08, 95.08	Triterpenoid	100
9	11.38	3,5,10-tris (acetyloxy)-2-hydroxy-4,14,16,16-tetramethyl-8-methylidene-13-oxo-15oxatetracyclo[9.4.1.0 ^1^, ^14^.0 ^4^, ^9^]hexadecan-7-yl 3-phenylprop-2-enoate	C _30_H _48_O _12_	639.28	621.27, 595.28, 579.26	Triterpenoid	59

### Isolation and structural elucidation of the isolated compounds

**Figure 3.**
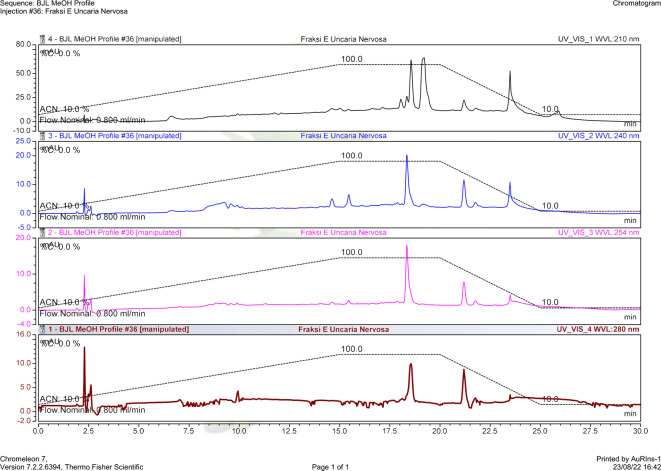
HPLC profile of *U. nervosa* Elmer subfraction E.

The ethanol extract of *U. nervosa* Elmer was separated using a fractionation method using n-hexane, ethyl acetate, and butanol as solvents. The isolation process was carried out on the ethyl acetate fraction using column chromatography. The column chromatography results obtained 11 subfractions, and based on monitoring the stain pattern using thin layer chromatography, the isolation was directed to subfraction E. The HPLC chromatogram of subfraction E can be seen in Figure 3.

**Figure 4.**
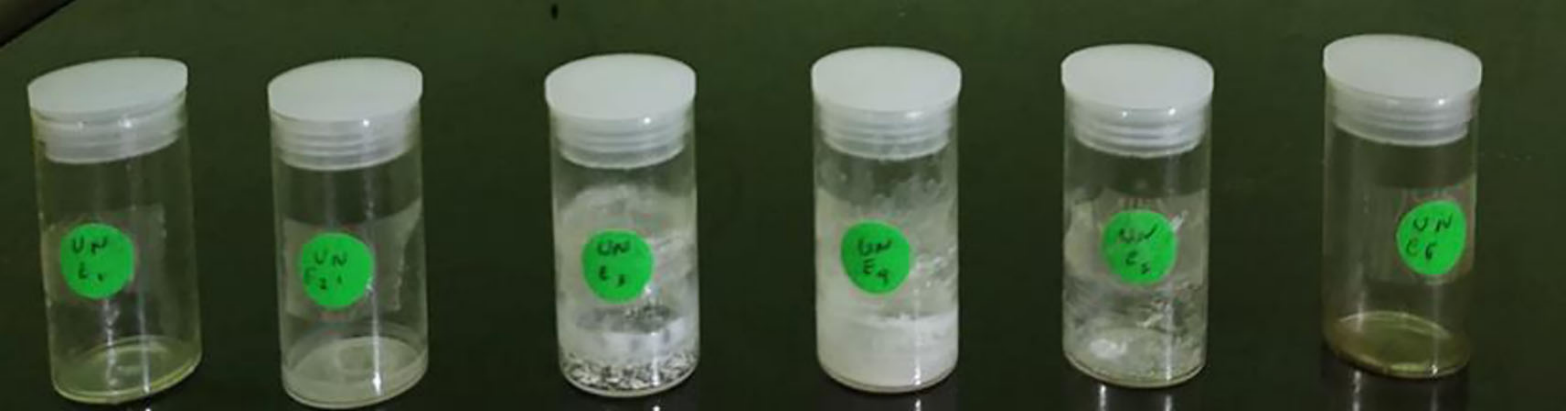
Six pure compounds from subfraction E.

**Figure 5.**
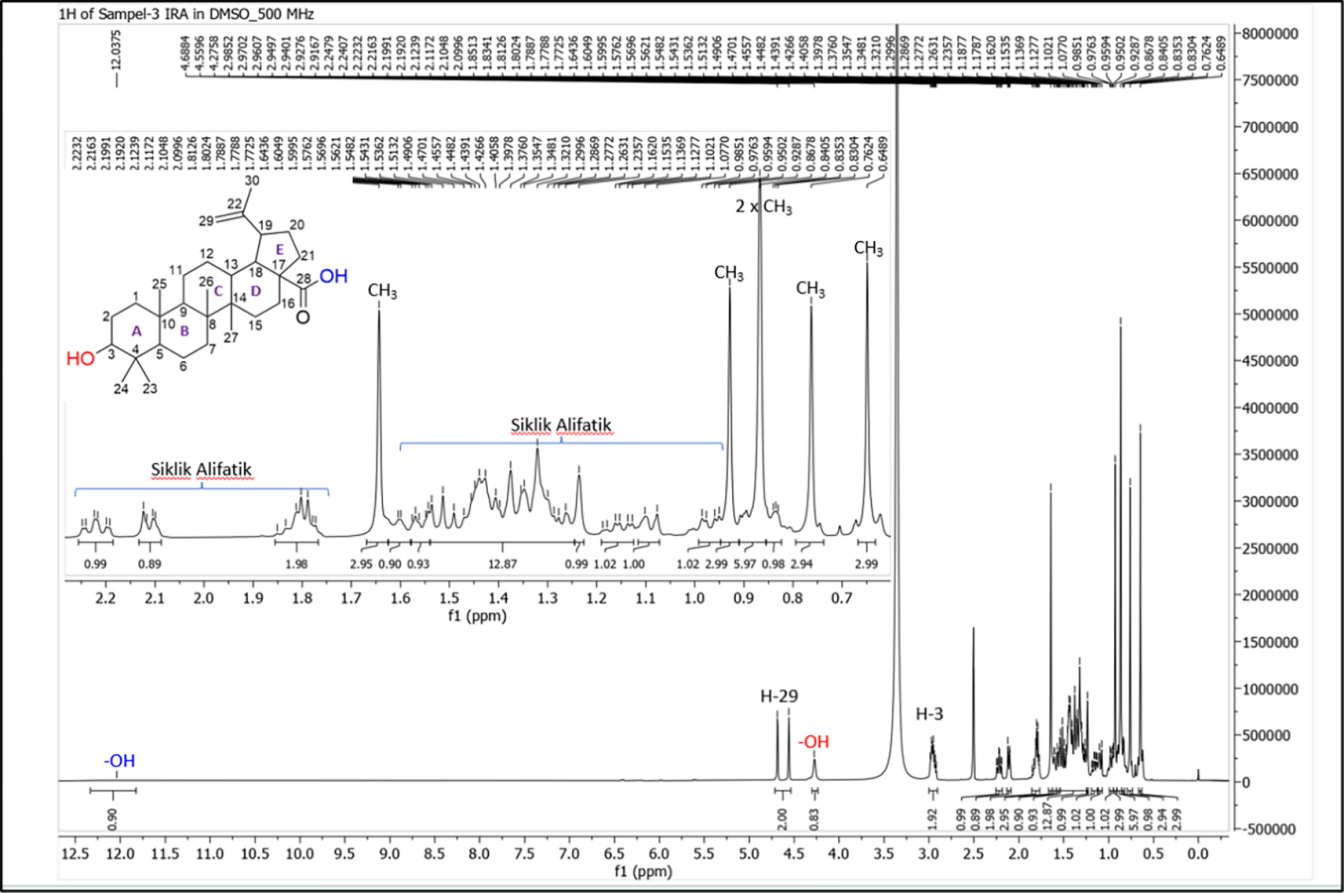
1H NMR compound UnE-3.

**Figure 6.**
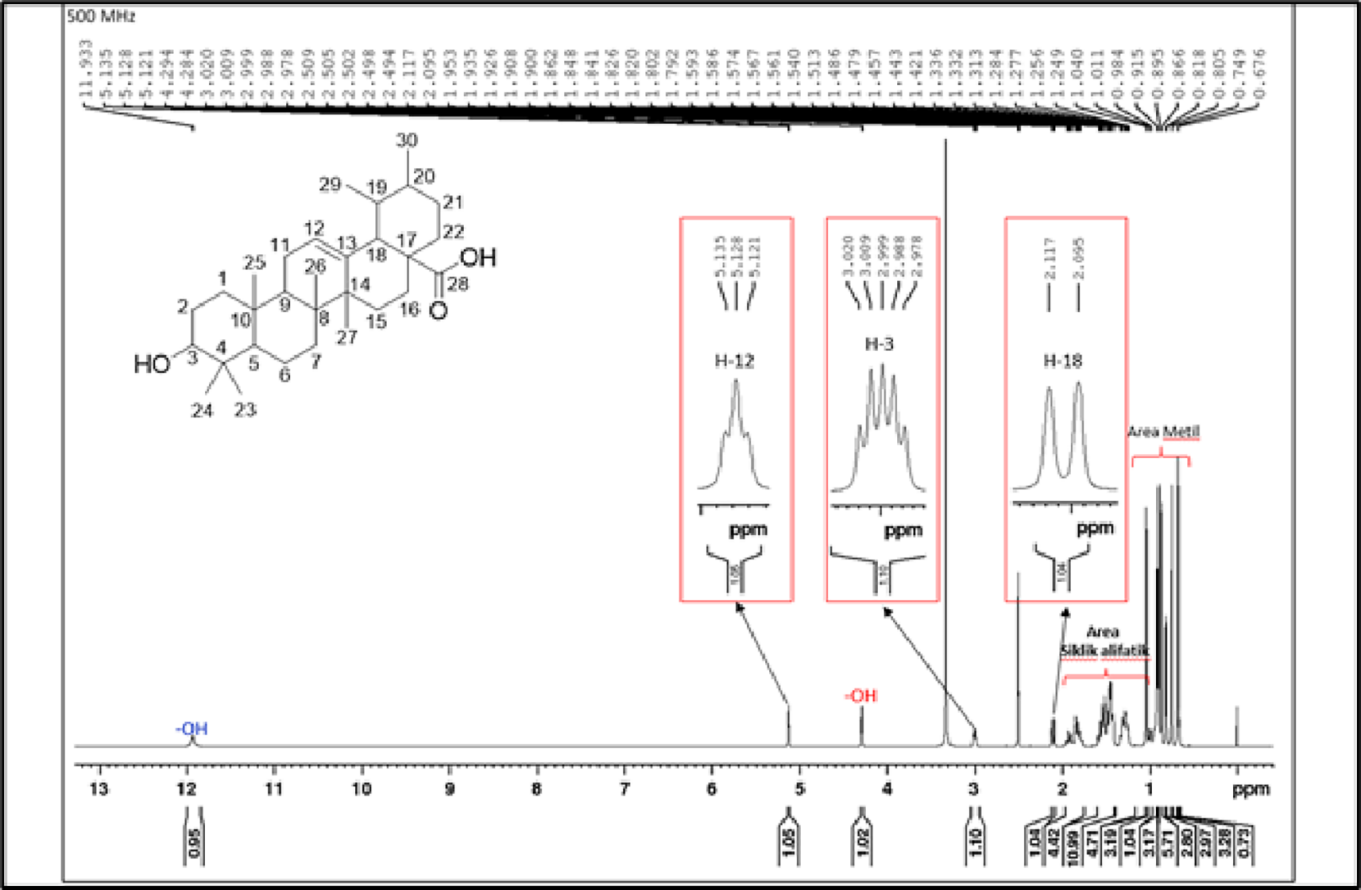
1H NMR compound UnE-4.

**Figure 7.**
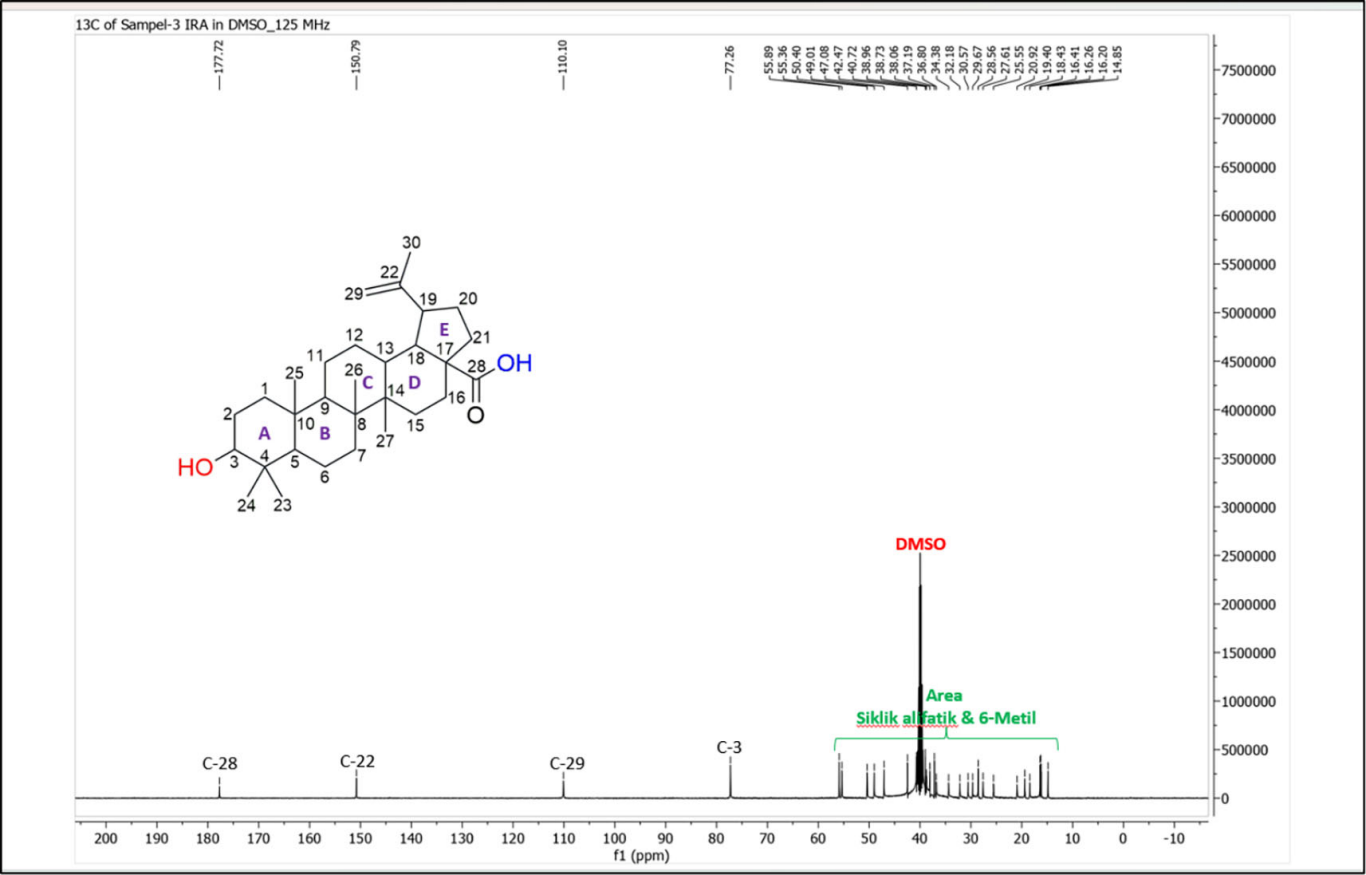
13 C NMR compound UnE-3.

**Figure 8.**
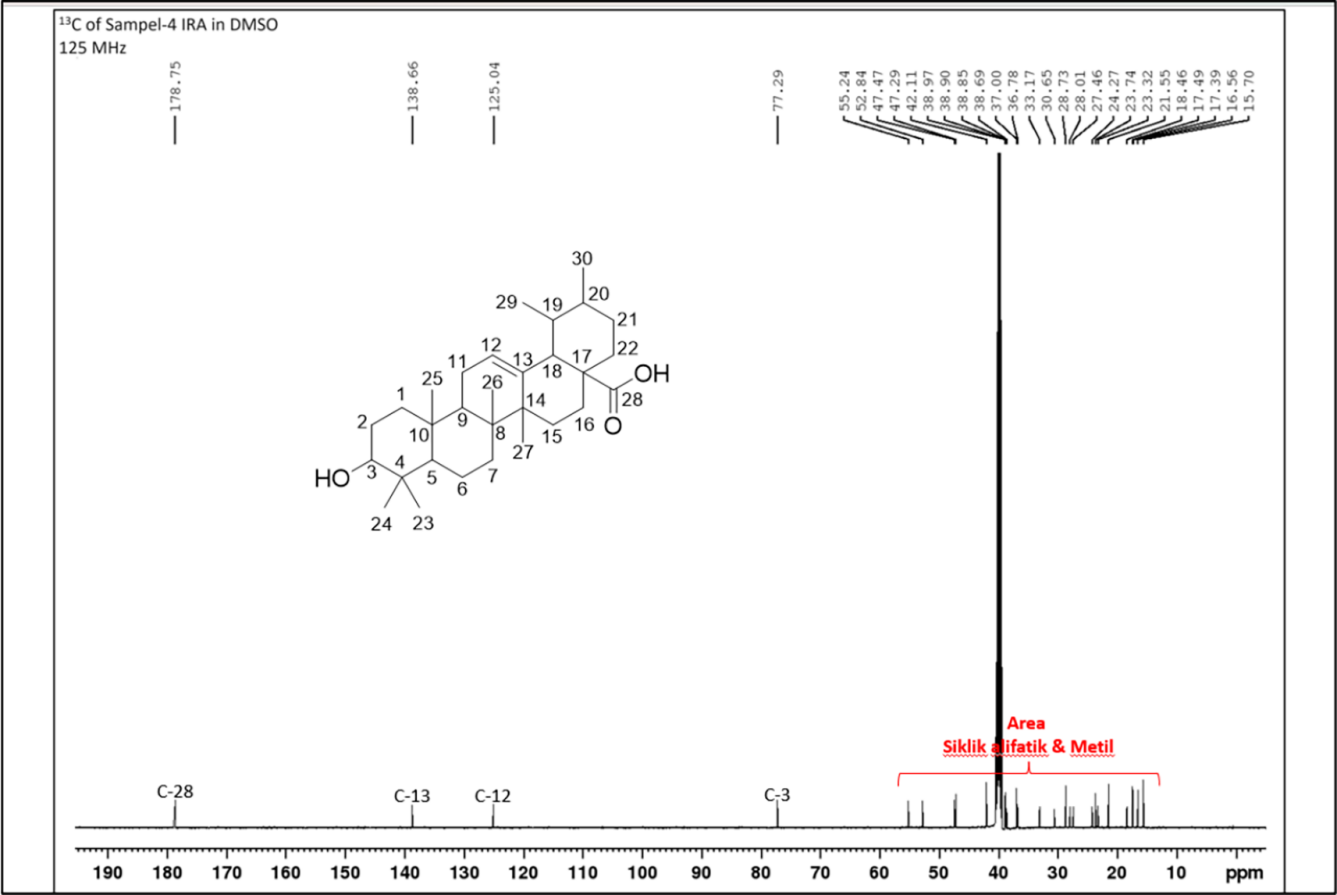
13 C NMR compound UnE-4.

**Figure 9.**
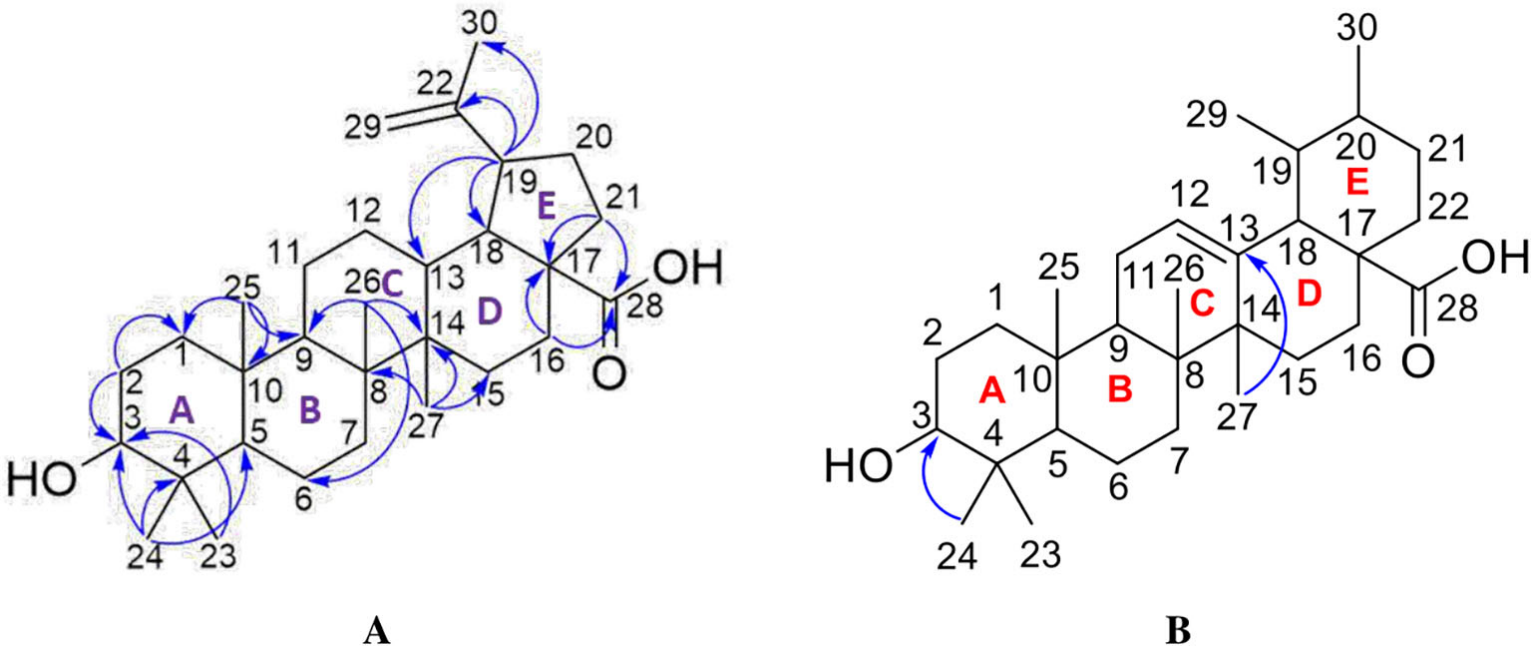
The structure of betulinic acid (A) and ursolic acid (B).

The isolation process for subfraction E was continued using a recycling preparative HPLC instrument, where the subfraction was dissolved in methanol and water using a C-18 column. The results of preparative HPLC recycling obtained six pure compounds ( Figure 4), and those that were elucidated were UnE-3 and UnE-4. Compounds Un-E-3 and UnE-4 were monitored by 1H and 13 C NMR, 1D NMR, and 2D NMR ( Figures 5, 6, 7, 8). The compounds that have been isolated are betulinic acid (UnE-3) and ursolic acid (UnE-4), with chemical structures as shown in Figure 9.

### Cytotoxic activity betulinic acid and ursolic acid against T47D cells

**Figure 10.**
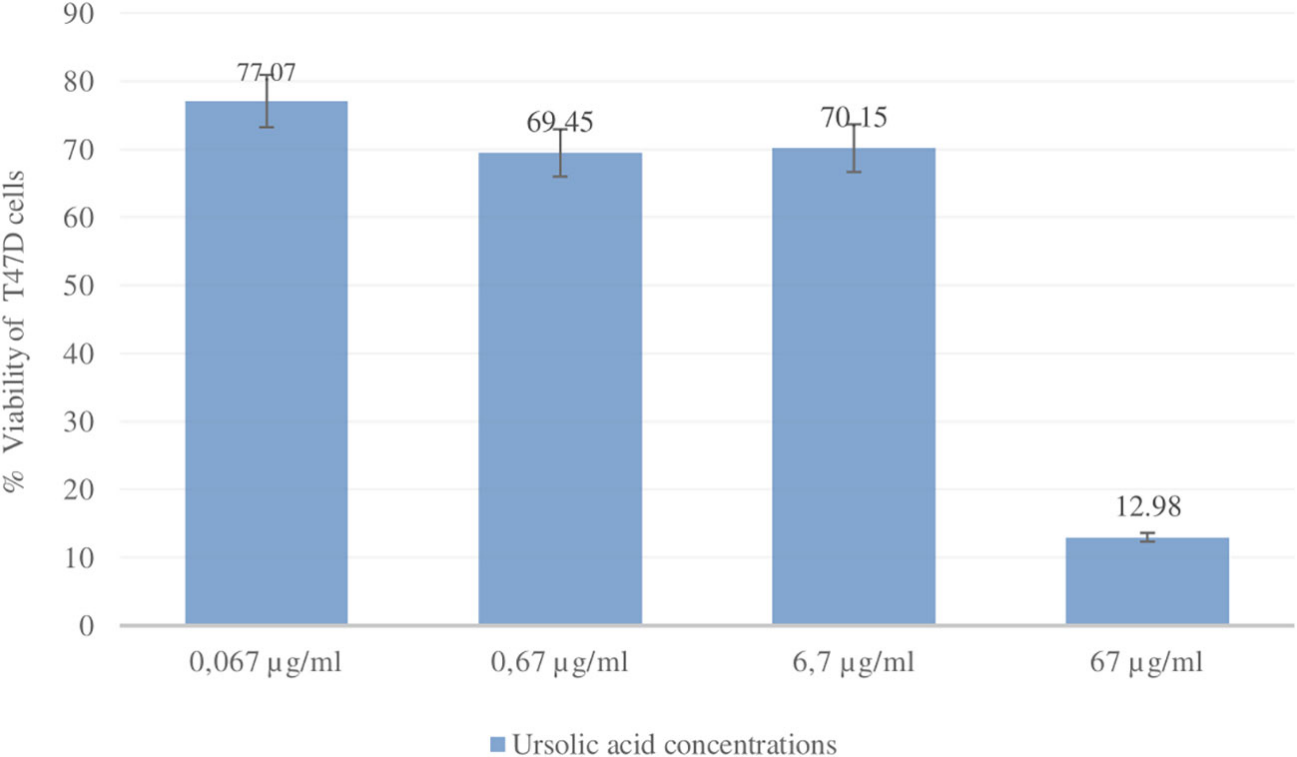
Graph of ursolic acid concentration (μg/ml) and % viability of T47D cells.

**Figure 11.**
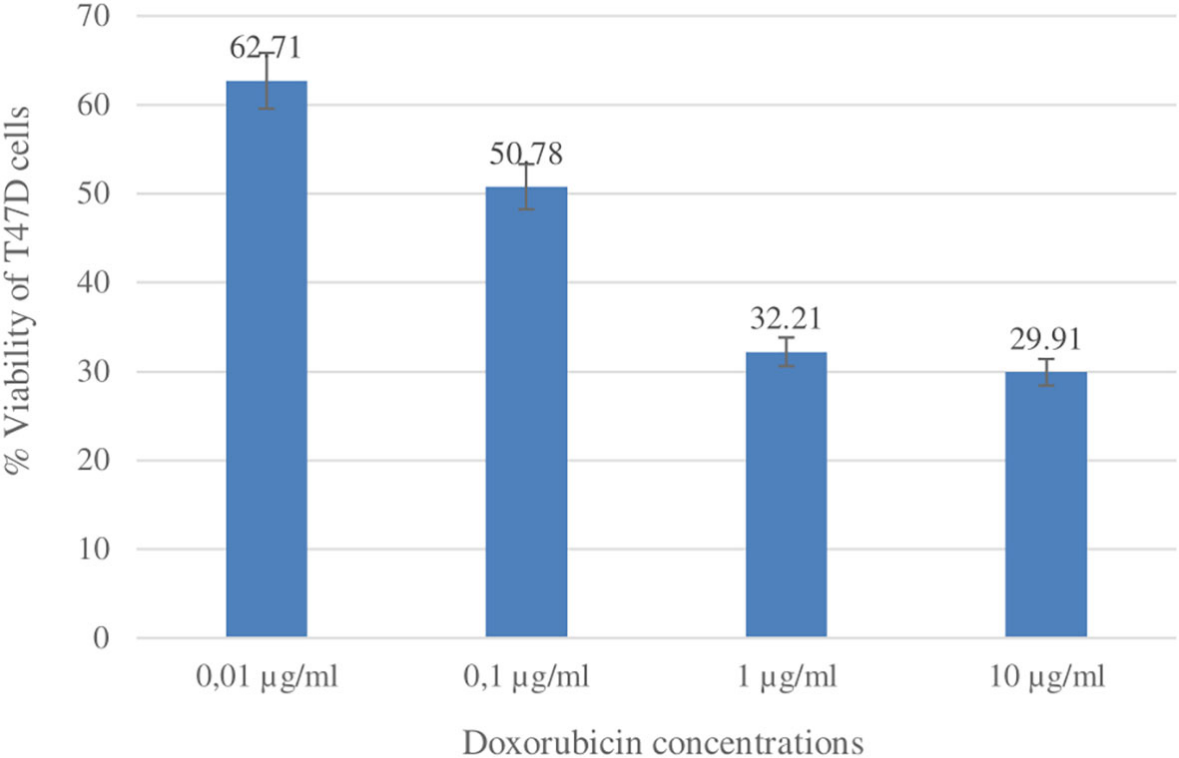
Graph of doxorubicin concentration (μg/ml) and % viability of T47D cells.

The cytotoxic activity of betulinic acid (BA) and ursolic acid (UA) on human breast cancer cells (T47D) was determined using the MTT method. As a positive control, the reference standard doxorubicin was used ( Numonov et al., 2020). Doxorubicin is one of the most widely used chemotherapy agents in the treatment of breast cancer. The results of the cytotoxic test showed that the IC _50_ values for UA and doxorubicin were 14.70 ± 4.50 μg/ml and 0.15 ± 0.02, respectively. The IC _50_ value for BA was not determined because the % viability at the highest concentration tested (53 μg/ml) was greater than 50%. The % viability values of UA and doxorubicin can be seen in Figures 10 and 11.

## Discussion

### Metabolites profiling of ethanol extract

Determining the metabolite profile of the extract begins with clean-up of the ethanol extract using the Solid Phase Extraction method (SPE). The extraction method that can be used for the analysis, separation, and purification of samples in the industrial, pharmaceutical, and toxicological fields. In this research, a cartridge containing a polyamide stationary phase was used. The stages carried out are conditioning, equilibration, sample loading, elution, evaporation of solvent, and yield recovery ( Abd Karim et al., 2022; Farré-Segura et al., 2022). Samples that have been cleaned up are prepared to determine the UHPLC profile and observed at 254 nm. In the chromatogram, it can be seen that in the ethanol extract, there are polar compounds with higher peaks than semi-polar and non-polar compounds. The metabolite profile of the ethanol extract was determined using LCMS/MS.

The ethanol extract data processing was carried out using the MZmine platform and compound identification using the Sirius platform ( Pang et al., 2024; Zheng et al., 2023). The ethanol extract is predicted to contain 9 compounds consisting of alkaloids, terpenoids, and fatty acids among them N-[(1,3-dimethyl-2,6-dioxo-7-prop-2-ynylpurin-8-yl) amino] formamide, N-(3-phenylbutyl) hexan-2-amine, 1,1-dichloro-1-nitrosopropane, ceratodictyol, betulinic acid, ursolic acid, 7-methyl-N-[6-[(7-methyl-6-oxooctanoyl) amino] hexyl]-6-oxononanamide, nervisterol, and 3,5,10-tris (acetyloxy)-2-hydroxy-4,14,16,16-tetramethyl-8-methylidene-13-oxo-15oxatetracyclo [9.4.1.0 ^1^, ^14^.0 ^4^, ^9^] hexadecan-7-yl 3-phenylprop-2-enoate. Based on the compounds predicted in the ethanol extract, there are 2 compounds that have been found in other species but have only just been discovered in this species, namely BA and UA. These two compounds are reported to have cytotoxic activity on several cancer cell ( Alam et al., 2021; Lou et al., 2021).

### Isolation and structural elucidation of the isolated compounds

The UnE-3 compound that has been isolated is a white powder. The molecular formula (C _30_H _48_O _3_) has an M/Z of 456,684 MS (-mode) along with its 1H NMR and 13C NMR spectral data. The compound has a UV spectrum that only has one peak at a maximum absorption of 195.18 nm. This indicates that this compound does not have a conjugated chromophore. To support the UV data, 1D NMR analysis (1H, 13C, and APT NMR in DMSO as well as 2D (HSQC and HMBC) solvent) was carried out. 13C NMR, 125 MHz analysis was carried out to see the total number of carbons. 30 signals were seen, which were divided into 4 types. chemical shift, namely 1 signal at δC 177.72 ppm carbonyl area, 2 signals at δC 150.79 and 110.10 ppm sp2 area, 1 signal at δC 77.26 ppm, and the remaining 26 signals in sp3 area. From the results of the analysis of all spectroscopic data and comparison with the literature, it can be concluded that this compound is classified as a pentacyclic triterpenoid, which has the name betulinic acid ( Rahmawati et al., 2024a).

BA is a pentacyclic triterpenoid compound and in *U. nervosa* leaves, it is the first to be reported. BA is a naturally occurring compound found primarily in the bark of certain trees, particularly in the white birch tree ( *Betula alba*). However, it’s not present in high concentrations in most plants. Apart from white birch, BA has also been found in other plants such as white or silver birch ( *Betula pendula*), sweet birch ( *Betula lenta*), yellow birch ( *Betula alleghaniensis*), white lupin ( *Lupinus albus*), *Syzygium* species ( Pai and Joshi, 2014), *Paronema canescens* ( Muharni et al., 2021), *Feretia canthioides* ( Egbubine et al., 2020), and *Tetracarpidium conophorum* ( Kelly et al., 2021).

The second compound that has been isolated is UnE-4, which is a white powder and has the molecular formula (C _30_H _48_O _3_) with m/z 456.66. This compound has a UV spectrum that only has one peak at a maximum absorption of 195.18 nm. This indicates that this compound does not have a conjugated chromophore. Furthermore, to support the UV data, 1D (1H and 13C NMR in DMSO solvent) and 2D (HMBC) NMR analysis was carried out. 13C NMR analysis, 125 MHz was carried out to see the total number of carbons which showed 30 signals which were divided into 4 types of chemical shifts, namely 1 signal at δC 178.75 ppm carbonyl area, 2 signals at δC 138.66 and 125.04 ppm sp2 area, 1 signal at δC 77.29 ppm and the remaining 26 signals in the sp3 area.

The 1H-NMR spectrum, 500 MHz there are several specific signals such as two hydroxy protons whose chemical environments are very different, namely at δH 11.93 (s, 1H) which usually bonds directly with the carbon sp2 carbonyl at C-28 while δH 4.29 (d, 1H) possibly binding to the sp3 carbon of methine at C-3. One sp2 area proton signals at δH 5.13 (t, J = 3.25 Hz, 1H) and the rest are aliphatic protons. HMBC data shows a long between H-24 and C-3 which confirms the position of the hydroxy methine which is in ring A. Furthermore, a long between H-27 and C-13 which turns off its position splits rings C and D. Based on Index calculations of Hydrogen Deficiency (IHD) obtained a value of 7 which is in good agreement with NMR data where 5 IHD comes from rings and 2 IHD from double bonds. From the results of the analysis of all spectroscopic data and comparison with the literature, it can be concluded that this compound is classified as a pentacyclic triterpenoid which has the name ursolic acid. UA has reportedly been isolated from several plants including *Salvia officinalis* ( Jedinák et al., 2006), *Apples* peels ( Yamaguchi et al., 2008), *Arbutus pavarii* ( Groshi et al., 2022), and *Uncaria macrophylla* ( Sun et al., 2012).

### Cytotoxicity

Compounds that have been isolated from the ethanol extract of *U. nervosa* Elmer leaves are BA and UA. These two compounds were subjected to a cytotoxic test on T47D breast cancer cells using the MTT method and doxorubicin was used as a positive control. The test results show that ursolic acid has strong activity when compared with betulinic acid. The IC _50_ values for ursolic acid and doxorubicin were 14.70 ± 4.50 μg/ml and 0.15 ± 0.02, respectively ( Rahmawati et al., 2024b). BA and UA are the active compounds of pentacyclic triterpenoids which is a triterpene acid that is found in various fruits, vegetables and medicinal plants ( Zafar et al., 2022; Maniyamma et al., 2022) and has been reported to have antidiabetic, anti-inflammatory, immunomodulatory, and anticancer activities ( Alam et al., 2021; Numonov et al., 2020; Sianipar, 2021; Wang et al., 2020). BA and UA are from natural products against metastasis and was also investigated in studies conducted in the last decade ( Khwaza et al., 2020; Naeem et al., 2022; Zafar et al., 2022).

Research related to the cytotoxic activity of BA includes the effects of skin carcinogenesis, where it was reported that BA can inhibit 70% of tumor development ( Agame-Lagunes et al., 2021). Research on MDA-MB-231 breast cancer cells gave an IC _50_ value of 38.81 ± 4.9 mg/mL ( Qi et al., 2021). BA was also reported to inhibit the viability of HuCCA and BHK-21 cells and induce neoplastic cell apoptosis ( Phonarknguen et al., 2022). Research related to the cytotoxic activity of UA has also been widely reported. UA is active in T47D breast cancer cells with an IC _50_ value of 1.63 ± 0.62 μM ( Numonov et al., 2020). UA increases cell sensitivity Triple-negative breast cancer (TNBC) to doxorubicin via signaling inactivation ZEB1-AS1/miR-186-5p/ABCC1 ( Lu et al., 2022). UA formulated into nanoparticles provides an IC _50_ value below 30 μM against pancreatic cancer cells AsPC-1 dan BxPC-3 ( Markowski et al., 2021) and UA can significantly increase the drug sensitivity of human breast cancer cells MCF-7/MDA-MB-231 against Epirubicin ( Wang et al., 2022).

## Conclusion

Betulinic acid, and Ursolic acid have been successfully isolated from leaves U. nervosa Elmer, and UA have moderate cytotoxic activity on T47D breast cancer cells.

## Ethical considerations

No animals were harmed in this research.

## Data Availability

Zenodo: (Data set) Isolation of Pentacyclic Triterpenoids from The Leaves of *Uncaria nervosa* Elmer. https://doi.org/10.5281/zenodo.11544271 This project contains the following underlying data:
• Dataset of this research Data are available under the terms of the Creative Commons Attribution 4.0 International license (CC4). Zenodo: (Data set) Cytotoxic activity of betulinic acid and ursolic acid againts T47D breast cancer cells. https://zenodo.org/doi/10.5281/zenodo.11545104 This project contains the following underlying data:
• Dataset of this research Data are available under the terms of the Creative Commons Attribution 4.0 International license (CCO).

## References

[ref1] Abd Karim HA Rasol NE Ismail NH : Metabolites annotation of dichloromethane extract of *Kibatalia maingayi* woods using Orbitrap High-Resolution Mass Spectrometry. *Malaysian J. Chem.* 2022;24:161–172. 10.55373/mjchem.v24i4.161

[ref2] Adham AN Hegazy MEF Naqishbandi AM : Induction of apoptosis, autophagy and ferroptosis by *Thymus vulgaris* and *Arctium lappa* extract in leukemia and multiple myeloma cell lines. *Molecules.* 2020;25:25. 10.3390/molecules25215016 33138135 PMC7663330

[ref32] Agame-Lagunes B Alegria-Rivadeneyra M Alexander-Aguilera A : Bioactivity of betulinic acid nanoemulsions on skin carcinogenesis in transgenic mice K14E6. *Grasas y Aceites.* 2021;72:e433. 10.3989/gya.0553201

[ref3] Alam M Ali S Ahmed S : Therapeutic potential of ursolic acid in cancer and diabetic neuropathy diseases. *Int. J. Mol. Sci.* 2021;22:22. 10.3390/ijms222212162 34830043 PMC8621142

[ref4] Ammar AS Ain N Abdull S : Mining secondary metabolites of *Chassalia curviflora* leaves using Sirius and High-Resolution Mass Spectrometry Data. *J. Sci. Mathem. Letters.* 2024;12:111–118. 10.37134/jsml.vol12.1.13.2024

[ref5] Anonim: Plants of the world online. n.d. Reference Source

[ref6] Bonsou IN Mbaveng AT Nguenang GS : Cytotoxicity, acute and sub-chronic toxicities of the fruit extract of Tetrapleura tetraptera (Schumm. & Thonn.) Taub. (Fabaceae). *BMC Complement. Med. Ther.* 2022;22:178. 10.1186/s12906-022-03659-1 35787267 PMC9252075

[ref7] Chooluck K Rojsanga P Phechkrajang C : Bioanalytical method validation for determination of rosmarinic acid in simulated biological media using HPLC. *Int. J. Appl. Pharm.* 2021;13:110–113. 10.22159/ijap.2021v13i2.40372

[ref33] Egbubine CO Adeyemi MM Habila JD : Isolation and characterization of betulinic acid from the stem bark of *Feretia canthioides* Hiern and its antimalarial potential. *Bull. Natl. Res. Cent.* 2020;44. 10.1186/s42269-020-00302-2

[ref8] El-Dawy K Mohamed D Abdou Z : Nanoformulations of pentacyclic triterpenoids: Chemoprevention and anticancer. *Int. J. Vet. Sci.* 2022;11:384–391. 10.47278/journal.ijvs/2021.100

[ref9] Farré-Segura J Le Goff C Lukas P : Validation of an LC-MS/MS method using Solid-Phase Extraction for the quantification of parathyroid hormone: Toward a candidate reference measurement procedure. *Clin. Chem.* 2022;68:1399–1409. 10.1093/clinchem/hvac135 36056745

[ref34] Groshi AA Nahar L MD Ismail F : Bioassay-guided isolation of ursolic acid as the major cytotoxic compound present in the methanolic extract of the leaves of *Arbutus pavarii* Pamp. *Dhaka Univ. J. Pharm. Sci.* 2022:20(3):267–274. 10.3329/dujps.v20i3.59792

[ref10] Hill RA Connolly JD : Triterpenoids. *Nat. Prod. Rep.* 2020;37:962–998. 10.1039/c9np00067d 32055816

[ref35] Jedinák A Mučková M Košt’álová D : Antiprotease and antimetastatic activity of ursolic acid isolated from *Salvia officinalis*. Zeitschrift Fur Naturforsch - Sect C. *J. Biosci.* 2006;61:777–782. 10.1515/znc-2006-11-1203 17294686

[ref11] Joshua JM Oyewale AO Ibrahim H : Isolation, characterization and antimicrobial screening of betulinic acid from the stem extract of *Fadogia erythrophloea.* *J. Chem. Soc. Niger.* 2020;45. 10.46602/jcsn.v45i4.492

[ref12] Kaps A Gwiazdoń P Chodurek E : Nanoformulations for delivery of pentacyclic triterpenoids in anticancer therapies. *Molecules.* 2021;26. 10.3390/molecules26061764 33801096 PMC8004206

[ref36] Kelly O Patrick OU Farzana S : Isolation, characterization, and hepatoprotective properties of betulinic acid and ricinine from *Tetracarpidium conophorum* seeds (Euphorbiaceae). *J. Food Biochem.* 2021;45. 10.1111/jfbc.13288 32529649

[ref37] Khwaza V Oyedeji OO Aderibigbe BA : Ursolic acid-based derivatives as potential anti-cancer agents: An update. *Int. J. Mol. Sci.* 2020;21:1–27. 10.3390/ijms21165920 32824664 PMC7460570

[ref29] Li Y Wang J Li L : Natural products of pentacyclic triterpenoids: from discovery to heterologous biosynthesis. *Nat. Prod. Rep.* 2023;40:1303–1353. 10.1039/d2np00063f 36454108

[ref13] Ling T Boyd L Rivas F : Triterpenoids as reactive oxygen species modulators of cell fate. *Chem. Res. Toxicol.* 2022;35:569–584. 10.1021/acs.chemrestox.1c00428 35312315 PMC9019815

[ref14] Lou H Li H Zhang S : A review on preparation of betulinic acid and its biological activities. *Molecules.* 2021;26. 10.3390/molecules26185583 34577056 PMC8468263

[ref15] Lu Q Chen W Ji Y : Ursolic acid enhances cytotoxicity of doxorubicin-resistant triple-negative breast cancer cells via ZEB1-AS1/miR-186-5p/ABCC1 Axis. *Cancer Biother. Radiopharm.* 2022;37:673–683. 10.1089/cbr.2020.4147 33493421

[ref39] Maniyamma A Ajesh V Uzini DD : Betulinic acid: A natural promising anticancer drug, current situation, and future perspectives. *J. Biochem. Mol. Toxicol.* 2022;36:23206. 10.1002/jbt.23206 36124371

[ref17] Markowski A Migdał P Zygmunt A : Evaluation of the in vitro cytotoxic activity of ursolic acid PLGA nanoparticles against pancreatic ductal adenocarcinoma cell lines. *Materials (Basel).* 2021;14. 10.3390/ma14174917 34501007 PMC8434451

[ref18] Maulina S Ryn Pratiwi D Erwin : Phytochemical screening and bioactivity of root extract of *Uncaria nervosa* Elmer (bajakah). 2019.

[ref40] Muharni F Yohandini H Napp FR : The Anticholesterol activity of betulinic acid and stigmasterol isolated from the leaves of sungkai ( *Paronema canescens* Jack.). *Int. J. App. Pharm.* 2021;13(2):198–203. 10.22159/ijap.2021v13i2.40372

[ref41] Naeem A Hu P Yang M : Natural Products as Anticancer Agents: Current Status and Future Perspectives. *Molecules.* 2022;27. 10.3390/molecules27238367 36500466 PMC9737905

[ref19] Newman DJ Cragg GM : Natural products as sources of new drugs over the nearly four decades from 01/1981 to 09/2019. *J. Nat. Prod.* 2020;83:770–803. 10.1021/acs.jnatprod.9b01285 32162523

[ref20] Noushahi HA Khan AH Noushahi UF : Biosynthetic pathways of triterpenoids and strategies to improve their biosynthetic efficiency. *Plant Growth Regul.* 2022;97:439–454. 10.1007/s10725-022-00818-9 35382096 PMC8969394

[ref42] Numonov S Sharopov F Qureshi MN : The ursolic acid-rich extract of *Dracocephalum heterophyllum* Benth. with potent antidiabetic and cytotoxic activities. *Appl. Sci.* 2020;10. 10.3390/APP10186505

[ref21] Pai SR Joshi RK : Distribution of betulinic acid in plant kingdom. *Plant Sci Today.* 2014;1:103–107. 10.14719/pst.2014.1.3.58

[ref22] Pang Z Xu L Viau C : MetaboAnalystR 4.0: a unified LC-MS workflow for global metabolomics. *Nat. Commun.* 2024;15:3675. 10.1038/s41467-024-48009-6 38693118 PMC11063062

[ref43] Phonarknguen R Nobsathian S Assawasuparerk K : Effect of betulinic acid extraction from Guava ( *Psidium guajava* Linn.) leaves against human cholangiocarcinoma cells. Asian Pacific. *J. Cancer Prev.* 2022;23:583–590. 10.31557/APJCP.2022.23.2.583 35225471 PMC9272617

[ref44] Qi X Gao C Yin C : Improved anticancer activity of betulinic acid on breast cancer through a grafted copolymer-based micelles system. *Drug Deliv.* 2021;28:1962–1971. 10.1080/10717544.2021.1979125 34565273 PMC8475105

[ref50] Rahmawati N Ismail NH Hamidi D : Cytotoxic Activity Screening of Various Uncaria spp Plants on T47d Breast Cancer Cells. *Trop. J. Nat. Prod. Res.* 2023;7:2218–2221. 10.26538/tjnpr/v7i1.19

[ref23] Rahmawati N Nor HI Fatma SR : Isolation of Pentacyclic Triterpenoids from The Leaves of *Uncaria nervosa* Elmer.(Dataset). *Zenodo.* 2024a. 10.5281/zenodo.11544271

[ref24] Rahmawati N Nor HI Fatma SR : Cytotoxic activity of betulinic acid and ursolic acid againts T47D breast cancer cells.(Dataset). *Zenodo.* 2024b. 10.5281/zenodo.11545104

[ref45] Sianipar EA . the Potential of Indonesian Traditional Herbal Medicine as immunomodulatory agents: a Review. Int. J. Pharm. Sci. Res. 2021;12:5229. 10.13040/IJPSR.0975-8232.12(10).5229-37

[ref47] Sun G Zhang X Xu X : A new triterpene from the plant of *Uncaria macrophylla*. *Molecules.* 2012;17:504–510. 10.3390/molecules17010504 22222909 PMC6268033

[ref26] Sung H Ferlay J Siegel RL : Global cancer statistics 2020: Globocan estimates of incidence and mortality worldwide for 36 cancers in 185 countries. *CA Cancer J. Clin.* 2021;71:209–249. 10.3322/caac.21660 33538338

[ref27] Wang C Gao Y Zhang Z : Ursolic acid protects chondrocytes, exhibits anti-inflammatory properties via regulation of the NF-κB/NLRP3 inflammasome pathway and ameliorates osteoarthritis. *Biomed. Pharmacother.* 2020;130:110568. 10.1016/j.biopha.2020.110568 32745911

[ref28] Wang Z Zhang P Jiang H : Ursolic acid enhances the sensitivity of MCF-7 and MDA-MB-231 cells to epirubicin by modulating the autophagy pathway. *Molecules.* 2022;27:1–13. 10.3390/molecules27113399 35684339 PMC9182048

[ref48] Yamaguchi H Noshita T Kidachi Y : Isolation of ursolic acid from apple peels and its specific efficacy as a potent antitumor agent. *J. Heal. Sci.* 2008;54:654–660. 10.1248/jhs.54.654

[ref49] Zafar S Khan K Hafeez A : Ursolic acid: a natural modulator of signaling networks in different cancers. *Cancer Cell Int.* 2022;22(1):399. 10.1186/s12935-022-02804-7 36496432 PMC9741527

[ref30] Zheng KX Liu CH Wang S : Evaluating the release and metabolism of ricinine from castor cake fertilizer in soils using a LC-QTOF/MS coupled with Sirius workflow. *Chemosphere.* 2023;310:136865. 10.1016/j.chemosphere.2022.136865 36244422

[ref31] Zhu Y Ouyang Z Du H : New opportunities and challenges of natural products research: When target identification meets single-cell multiomics. *Acta Pharm. Sin. B.* 2022;12:4011–4039. 10.1016/j.apsb.2022.08.022 36386472 PMC9643300

