# Supplementary material for: Uncaria nervosa Elmer, a new herbal source for betulinic acid and ursolic acid: Metabolites profiling, isolation, and in vitro cytotoxicity studies against T47D breast cancer
Source: F1000Res. 2025 Sep 15;13:923. Originally published 2024 Aug 15. [Version 2] doi: 10.12688/f1000research.152293.2 (PMC12550495; doi:10.12688/f1000research.152293.2)
Supplement: Peer review history

# Reviewer report: Reviewer response for version 2

**DOI:** 10.5256/f1000research.186569.r414395

**Published:** 2025-10-23

**Authors:** Suhuan Mei

**Affiliations:** 1 Bioscience and Engineering, Jiangxi Agricultural University, Nanchang, Jiangxi, China

The authors have thoroughly addressed the reviewers' comments and made improvements in the revised manuscript. I recommend acceptance.

Is the work clearly and accurately presented and does it cite the current literature?

Yes

If applicable, is the statistical analysis and its interpretation appropriate?

Yes

Are all the source data underlying the results available to ensure full reproducibility?

Yes

Is the study design appropriate and is the work technically sound?

Yes

Are the conclusions drawn adequately supported by the results?

Yes

Are sufficient details of methods and analysis provided to allow replication by others?

Yes

Reviewer Expertise:

1. Separation, characterization, and evaluation of bioactive components from foods or plants. 2. The impact of natural products on gut health. 3. Safety evaluation of natural products. 4. Chemical and functional changes of foods during processing

I confirm that I have read this submission and believe that I have an appropriate level of expertise to confirm that it is of an acceptable scientific standard.

# Reviewer report: Reviewer response for version 2

**DOI:** 10.5256/f1000research.186569.r414394

**Published:** 2025-10-16

**Authors:** Subiya Showkat

**Affiliations:** 1 Bharathidasan University, Tiruchirappalli, Tamil Nadu, India

The authors have revised the manuscript as per my previous comments. I have no further suggestions, and the manuscript may be considered for indexing.

Is the work clearly and accurately presented and does it cite the current literature?

Partly

If applicable, is the statistical analysis and its interpretation appropriate?

Yes

Are all the source data underlying the results available to ensure full reproducibility?

Yes

Is the study design appropriate and is the work technically sound?

Yes

Are the conclusions drawn adequately supported by the results?

No

Are sufficient details of methods and analysis provided to allow replication by others?

No

Reviewer Expertise:

Phytochemistry, pharmacology, Microbiology and computational studies.

I confirm that I have read this submission and believe that I have an appropriate level of expertise to confirm that it is of an acceptable scientific standard.

# Reviewer report: Reviewer response for version 1

**DOI:** 10.5256/f1000research.167034.r320017

**Published:** 2025-03-07

**Authors:** Suhuan Mei

**Affiliations:** 1 Bioscience and Engineering, Jiangxi Agricultural University, Nanchang, Jiangxi, China

This paper illustrates the cytotoxicity against T47D breast cancer of betulinic acid and ursolic acid from *Uncaria nervosa Elmer*. The authors have performed LC-MS, UV, HPLC, NMR, and 2D NMR to isolate and characterize bioactive compounds as well as the MTT method to evaluate the cell viability. The rationale of the proposal is logical and the manuscript is written well but the manuscript should be revised considering the following points which should be incorporated for its improvement.

 1. In the title, 'Elmer' does not need italics.

 2. The weight of 1.932 g leaves is dry weight or wet weight should be described in the section of methods. Please address the manuscript thoroughly.

 3. Details of the NMR should be described in the method section.

 4. Is there any data about the yield of the different fractions and pure compounds?

 5. Results and discussion have many similar descriptions, for example,

 “Results

 Metabolites profiling of ethanol extract

 Determination of the metabolite profile of ethanol extract begins with the clean-up process of ethanol extract using the solid phase extraction (SPE) method.”

 “Discussion

 Metabolites profiling of ethanol extract

 Determining the metabolite profile of the extract begins with clean-up of the ethanol extract using the Solid Phase Extraction method (SPE).”

 6. An acronym of the full name is necessary when they appear first.

 7. Please don't use the same subheading in the result and discussion.

 8. The sentence 'The ethanol extract data processing was carried out using the MZmine platform and compound identification using the Sirius platform (Pang et al., 2024; Zheng et al., 2023)' in the discussion seems more appropriate when it appears in the method. 

 9. As mentioned above, please check the manuscript carefully, don’t repeat the result, and add more in-depth discussions.

 10. Is the ability of betulinic acid and ursolic acid from Uncaria nervosa Elmer any different compared with other herbal plants?

Is the work clearly and accurately presented and does it cite the current literature?

Yes

If applicable, is the statistical analysis and its interpretation appropriate?

Yes

Are all the source data underlying the results available to ensure full reproducibility?

Yes

Is the study design appropriate and is the work technically sound?

Yes

Are the conclusions drawn adequately supported by the results?

Yes

Are sufficient details of methods and analysis provided to allow replication by others?

Yes

Reviewer Expertise:

1. Separation, characterization, and evaluation of bioactive components from foods or plants. 2. The impact of natural products on gut health. 3. Safety evaluation of natural products. 4. Chemical and functional changes of foods during processing

I confirm that I have read this submission and believe that I have an appropriate level of expertise to confirm that it is of an acceptable scientific standard, however I have significant reservations, as outlined above.

# Response

**Published:** 2025-03-17

**Authors:** Noveri Rahmawati

**Affiliations:** Pharmacy, Universitas Andalas, Padang, West Sumatra, Indonesia

Thank you very much for all the comments from the reviewers. We will thoroughly revise this article and send it back to the editor. This is a response to the reviewer's comments.

**1**. Thank you. We will revise it.

**2**. The weight of 1.932 g leaves is dry weight. We will revise it. Thank you.

**3**. We will include the NMR in the method. Thank you.

**4**. We have data on the results of different fractions and pure compounds. We will include them in the article.

**5**. Thank you. We will revise it.

**6**. Thank you. We will revise it.

**7**. Thank you. We will revise it.

**8**. Thank you. We will revise it.

**9**. Thank you. We will revise it.

**10**.The ability of betulinic acid and ursolic acid from Uncaria nervosa Elmer on cancer cells is no different when compared to other herbal plants. Betulinic acid and ursolic acid have been reported to have potent bioactivity on several T47D breast cancer cells.

# Reviewer report: Reviewer response for version 1

**DOI:** 10.5256/f1000research.167034.r364783

**Published:** 2025-02-18

**Authors:** Subiya Showkat

**Affiliations:** 1 Bharathidasan University, Tiruchirappalli, Tamil Nadu, India

After carefully reviewing the manuscript and providing my comments, I believe that the manuscript has potential but requires substantial revisions. I recommend that the authors address the issues raised before the manuscript can be reconsidered for indexing.

**Comments for authors:**

1. In the introduction, several statements are made about the medicinal properties of *U.* *nervosa*, such as its wide range of health benefits and its traditional use by indigenous communities *. U. nervosa* has been revered for its diverse medicinal properties and has been used by indigenous communities for various health purposes. In traditional herbal medicine, different parts of the plant, including the leaves, stems, and roots, are utilized to prepare decoctions, infusions, or poultices to address ailments such as fever, inflammation, digestive disorders, and respiratory conditions. Additionally, *U.* *nervosa* has been valued for its purported benefits in promoting overall wellness and vitality. However, there is no reference provided to support these claims. It would be helpful to include appropriate citations to validate the medicinal properties attributed to the plant.
2. In the methods section, the ethyl acetate fraction is mentioned as being weighed and then subjected to chromatography. However, in the results, it mentions that the ethanol extract was fractionated and then the ethyl acetate fraction was used for isolation. This creates some confusion. Could you clarify whether the isolation was performed on the ethanol extract or the ethyl acetate fraction? Additionally, 20 g of the ethyl acetate fraction was obtained, which seems questionable, as 20 g appears too large. Was this fraction liquid? If so, was it dried before weighing?
3. There are many places in the manuscript where the grammar should be checked. Additionally, there are several sentences that are not well connected.
4. What was the rationale for choosing ethanol extract of *U. nervosa* for analysis in this study, and how does it compare to other possible extraction methods or solvents in terms of efficiency and bioactivity?"
5. The methodology for NMR studies is missing.
6. The conclusion is very short and not satisfactory.
7. The results and discussion section seems very weak and lacking in depth.
8. Have the authors used a standard for the HPLC analysis? If so, it should be properly labeled in the figure.
9. Ensure all scientific names are italicized throughout the manuscript.

Is the work clearly and accurately presented and does it cite the current literature?

Partly

If applicable, is the statistical analysis and its interpretation appropriate?

Yes

Are all the source data underlying the results available to ensure full reproducibility?

Yes

Is the study design appropriate and is the work technically sound?

Yes

Are the conclusions drawn adequately supported by the results?

No

Are sufficient details of methods and analysis provided to allow replication by others?

No

Reviewer Expertise:

Phytochemistry and pharmacology.

I confirm that I have read this submission and believe that I have an appropriate level of expertise to confirm that it is of an acceptable scientific standard, however I have significant reservations, as outlined above.

# Response

**Published:** 2025-03-17

**Authors:** Noveri Rahmawati

**Affiliations:** Pharmacy, Universitas Andalas, Padang, West Sumatra, Indonesia

Thank you for all the suggestions for our article. We will revise the article thoroughly and send it back to the editor. Herewith we convey some responses to the reviewer's comments.

**1**. Thank you. We will provide references related to the benefits of plants.

**2**. Isolation was carried out on the ethyl acetate fraction. Ethanol extract 70% (514 g) was fractionated in stages using n-hexane, ethyl acetate and butanol solvents. The dry ethyl acetate fraction obtained was 20 g.

**3**. Thank you. We will revise it.

**4**. **The basis for selecting ethanol:** ethanol is a polar solvent (lower polarity than water) so it can attract bioactive compounds found in *U. nervosa* elmer leaves such as alkaloids, flavonoids, and phenolics.

**Comparison with other extraction methods or solvents in terms of efficiency and bioactivity:**

 Extraction using the maceration method is a simple extraction without heating so it is more efficient and allows the bioactive compounds contained in plants to remain stable.

 The use of other solvents such as water will only be able to attract polar compounds and is less effective for lipophilic compounds.

 Methanol solvents can extract phenolic compounds more strongly, but are more toxic than ethanol so they are less suitable for biomedical applications.

 n-hexane and dichloromethane solvents will be effective in attracting non-polar compounds such as essential oils.

**5**. Thank you. We will revise it.

**6**. Thank you. We will revise it.

**7**. Thank you. We will revise it.

**8**. Yes, we use standards in HPLC analysis and we will label them correctly in the images. Thank you.

**9**. Thank you. We will revise it.
